# Damage-free peripheral nerve stimulation by 12-ns pulsed electric field

**DOI:** 10.1038/s41598-017-10282-5

**Published:** 2017-09-05

**Authors:** Maura Casciola, Shu Xiao, Andrei G. Pakhomov

**Affiliations:** 1 Frank Reidy Research Center for Bioelectrics, Old Dominion University, Norfolk, VA 23508 USA; 2 Department of Electrical and Computer Engineering, Old Dominion University, Norfolk, VA 23508 USA

## Abstract

Modern technologies enable deep tissue focusing of nanosecond pulsed electric field (nsPEF) for non-invasive nerve and muscle stimulation. However, it is not known if PEF orders of magnitude shorter than the activation time of voltage-gated sodium channels (VGSC) would evoke action potentials (APs). One plausible scenario requires the loss of membrane integrity (electroporation) and resulting depolarization as an intermediate step. We report, for the first time, that the excitation of a peripheral nerve can be accomplished by 12-ns PEF without electroporation. 12-ns stimuli at 4.1–11 kV (3.3–8.8 kV/cm) evoked APs similarly to conventional stimuli (100–250 μs, 1–5 V, 103–515 V/m), except for having higher selectivity for the faster nerve fibers. Nerves sustained repeated tetanic stimulations (50 Hz or 100 Hz for 1 min) alternately by 12-ns PEF and by conventional pulses. Such tetani caused a modest AP decrease, to a similar extent for both types of stimuli. Nerve refractory properties were not affected. The lack of cumulative damages even from tens of thousands of 12-ns stimuli and the similarities with the conventional stimulation prove VGSC activation by nsPEF without nerve membrane damage.

## Introduction

In past decades, neurostimulation has gained attention in medical applications where conventional pharmacological approaches become ineffective or are not tolerated by patients. Neurostimulation helped patients with refractory pain, Parkinson disease, dystonia, psychiatric and mood disorders, epilepsy, bladder dysfunction, and other conditions^1–7^.

Recent advancements in pulsed power physics and engineering have extended the range of electric stimuli to nano- and sub-nanosecond pulse durations^8–14^. Such stimuli can be focused deep in tissues without inserting electrodes, thereby opening tremendous opportunities for targeted non-invasive electrostimulation. However, little is known if such brief stimuli are suitable at all for electrostimulation. Simple scaling of the strength-duration curves for electrostimulation [26,27] into the nsPEF range is questionable because of: (1) a qualitatively different physical mechanism of cell membrane polarization by nsPEF, (2) the time needed for the transition of VGSC into the “open” conformation, and (3) the possibility of concurrent membrane damage by electroporation.

Specifically, conventional electrostimulation by milli- and microsecond pulses relies on the movement of ions to build up a high potential difference at the membrane interface. This process, known as Maxwell-Wagner ionic polarization, amplifies the externally applied electric field more than 1,000-fold, so that the transmembrane potential threshold can be reached even with low voltage stimuli. Cell membrane depolarization at the cathode-facing pole of the cell leads to VGSC opening and AP firing.

However, the amplifying effect of the ionic polarization tapers out as the pulse duration drops to singe nanoseconds, by giving little time for ions to migrate towards the membrane. Still, the applied field is amplified by a dielectric stacking effect, but only 5–20-fold (see ref. 12 for discussion), and the relaxation of the induced transmembrane potential is essentially instant. VGSC opening is effected by a shift of the voltage sensor of the channel, which, under physiological conditions, takes tens to hundreds of microseconds; it is not known if such a shift can occur in only a few nanoseconds even if the electric field is made infinitely high. On the contrary, molecular modeling simulations show that hydrophilic pore formation in a lipid bilayer occurs in nanoseconds^15, 16^. Indeed, multiple experimental studies have demonstrated long-lasting injury to cell membrane by nsPEF^9, 11, 13, 14, 17–20^, but reports about electrostimulation by nsPEF are scarce and contradictory.

Rogers and co-authors were the first to stimulate an isolated frog gastrocnemius muscle with pulses down to 1 ns duration^21^. The excitation was judged by a muscle twitch and was assumed to result from stimulation of presynaptic nerve terminals by nsPEF. The threshold for 1-ns pulses (24 kV/cm) fell reasonably well on the strength-duration curve, which spanned down from millisecond durations. Therefore, the authors suggested that the mechanism of excitation by nsPEF was not different from longer stimuli, but did not discuss or prove this conjecture.

Two studies from Cooper’s group compared excitation and electroporation thresholds in isolated nociceptor neurons^22, 23^. The excitation thresholds reported in these studies are exceptionally low (down to 0.4 kV/cm for a single 12-ns PEF and 0.016 kV/cm for a brief train), whereas the neuronal injury (judged by the uptake of propidium dye) required the electric field 30–50 times higher.

A recent study in cultured hippocampal neurons^24^ established similar thresholds for excitation and electroporation by 200-ns pulses (1.9–4 and 1.5–1.9 kV/cm, respectively). Despite this similarity, a comparison of the fast kinetics of nsPEF-induced membrane potential change, with and without tetrodotoxin, demonstrated that opening of VGSC was not mediated by the loss of the membrane potential due to electroporation. The study suggested a low potency of nsPEF, as compared to conventional electrostimulation, for VGSC activation and AP induction.

In adult rat cardiomyocytes, the threshold for Ca^2+^ mobilization by 4-ns pulses was at 10–80 kV/cm^25^. Although the authors did not exclude direct activation of voltage-gated calcium channels (VGCC) by nsPEF, they thought that it was mediated by cell membrane electroporative injury. The injury caused leak currents and a prolonged depolarization, which was more than sufficient to trigger VGCC opening. Studies in embryonic rat cardiomyocytes established a similar threshold (36 kV/cm) for eliciting Ca^2+^ transients by 10-ns PEF, whereas the electroporation threshold was about twofold higher (63 kV/cm)^11^. With 0.5-ns, 190 kV/cm PEF, Ca^2+^ mobilization in different cell types was always mediated by VGCC opening, and the results were indicative of a non-conventional membrane electroporation which involves membrane proteins rather than just lipids^12^. In bovine chromaffin cells, a single 5-ns, 50 kV/cm PEF caused Ca^2+^ mobilization which depended on L-type VGCC and was mediated by the tetrodotoxin-insensitive Na^+^ uptake; the data argued against direct activation of VGSC by nsPEF^26^. Other studies with 10-ns pulses did not even consider possible activation of VGSC and regarded Ca^2+^ mobilization solely as a result of electroporation^27^.

This question whether nsPEF can activate voltage-gated channels directly, without electroporative injury, is of key importance for medical applications of nsPEF stimulation. However, the above studies provided no definitive answer, possibly because the experiments were performed in different objects and the complexity of nsPEF effects was underestimated. Voltage-gated channels in living cells are under a tight regulation by diverse signaling cascades, which may be a separate target for nsPEF^28, 29^, thereby smearing the results. Furthermore, externally-applied PEF depolarizes one pole of the cell but hyperpolarizes the opposite pole; the opening of hyperpolarization-activated channels may prevent excitation. For conventional (long) stimuli, the interplay of de- and hyperpolarization led to a peculiar “window” effect^30^ (i.e., excitation within only a certain range of pulse amplitudes). However, it is not known how such effect would translate to nsPEF and to different types of cells, with different sets of endogenous ion channels.

Peripheral nerves are composed of axons of somatosensory and motor neurons; they are intended primarily for a robust long-distance AP conduction and lack the complexity of the whole cell. Peripheral nerve fibers operate in the “all-or-none” mode, which is not known to be modulated by intracellular signaling. Nerve fibers have a limited and well-known selection of ion channels and transporters and arguably are among the simplest excitable structures. Large linear dimensions of the nerve allow for spatial separation of de- and hyperpolarized areas, so that the latter will not interfere with AP initiation at the former. These features made isolated peripheral nerves a convenient model for studying effects of various physical factors on excitation^31^. Finally, peripheral nerves will likely be a target for various neuromodulation therapies, but they have never been tested for excitation by nsPEF.

Here we show, for the first time, that 12-ns PEF can elicit thousands of APs without damage to nerve fibers. The exact mechanism how nsPEF activates VGSC remains to be elucidated; however, it clearly does not involve electroporation as an intermediate step.

## Materials and Methods

### Nerve preparation

All experiments were performed in isolated nerve preparations (*n. ischiadicus* + *n. peroneus*) of the frog *Xenopus laevis*. Active adult frogs (males) were housed in aquariums following standard protocols. Animals were euthanized by pithing the brain and the spinal cord, then nerves from both legs were isolated from surrounding tissues, ligated and submerged in a chilled physiological solution containing (in mM): 140 NaCl, 5.4 KCl, 1.5 MgCl_2_, 2 CaCl_2_, 10 Glucose, and 10 HEPES (pH 7.3, 290–300 mOsm/kg, 1.6 S/m). All animal procedures were approved by the Old Dominion University Institutional Animal Care and Use Committee. All experiments were performed in accordance with relevant guidelines and regulations.

Experiments were conducted at room temperature. Each nerve preparation underwent several experiments over a period of 4–5 hours. For any experimental protocol, treatment parameters were varied in a random fashion, and each protocol was tested repeatedly in different nerve preparations. The mean data values for different groups were compared using an unpaired two-tailed *t*-test. Data are presented in graphs as mean values ± standard error for *n* independent experiments; p < 0.05 was considered statistically significant. The graphs were generated with Grapher 11 (Golden Software, Golden, CO).

### Nerve stimulation and recording of compound action potentials (CAPs)

**Figure 1:**
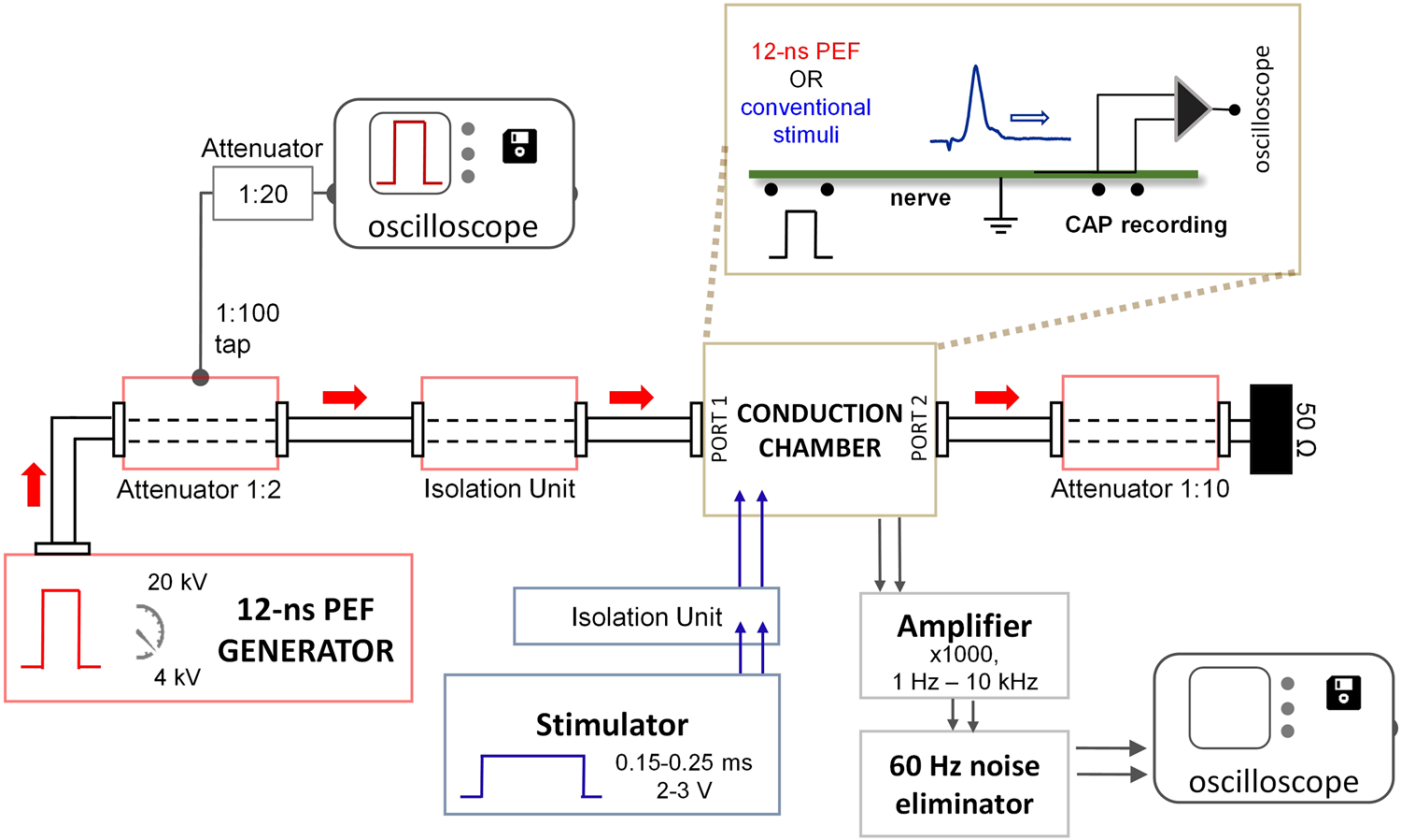
Block diagram of the setup for 12-ns PEF stimulation. Isolated nerve is placed in the conduction chamber which permits switching between 12-ns PEF (red arrows) and conventional stimulation (blue arrows). See Fig. 2 and text for more details.

**Figure 2:**
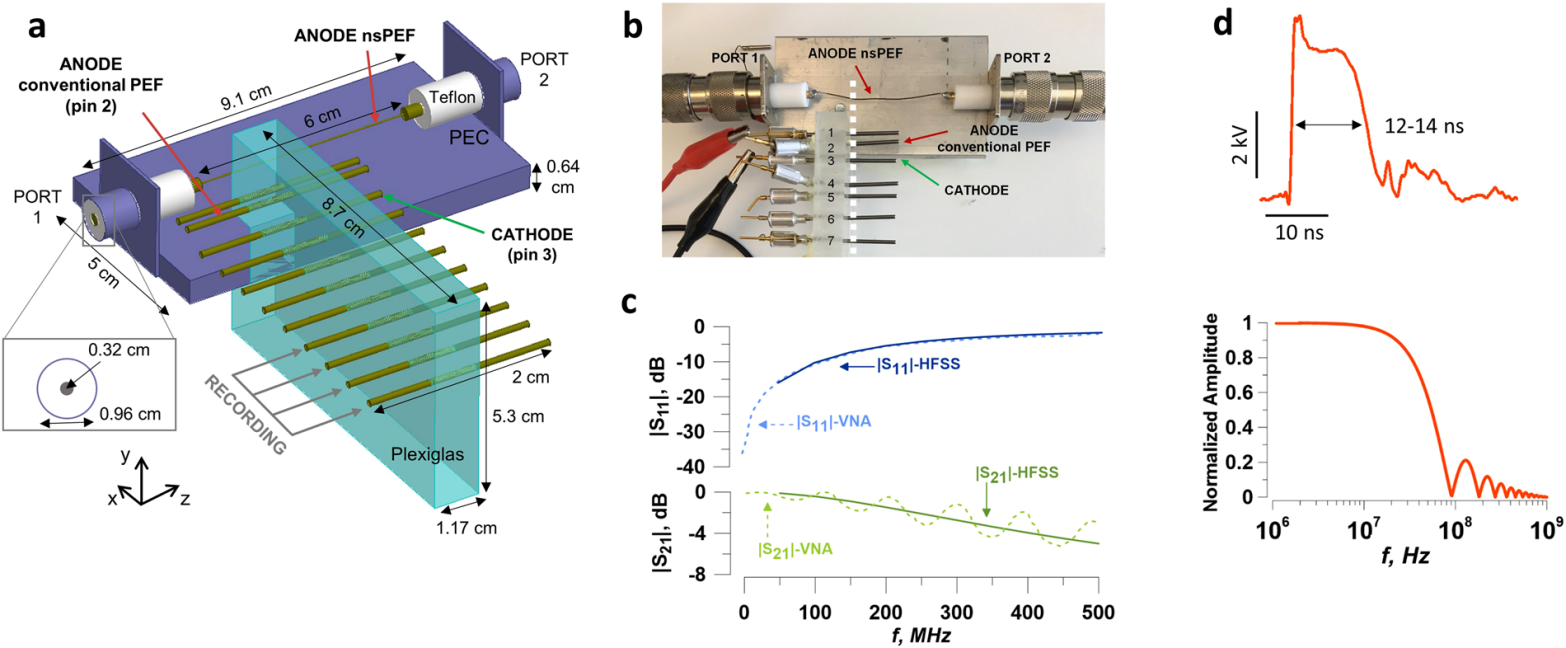
Conduction chamber design and electric stimulation of the nerve. (**a**) Schematic of the conduction chamber. The nerve (not shown) is placed atop the metal pins and the open central conductor of the coaxial line. (**b**) A photo of the chamber, with the approximate position of the nerve shown by a dashed line. (**c**) Scattering parameters of the conduction chamber. Modules of the reflection and transmission coefficients (|S_11_| and |S_21_|, respectively) over frequency as obtained from HFSS simulations (solid lines) and from VNA measurements (dashed lines). (**d**) The shape of the 12-ns pulse (top), with the width of 12–14 ns at 50% height, and the spectrum of an ideal pulse used for simulations (10 ns plateau, 1 ns rise and fall times).

Figure 1 shows a block diagram of the experimental setup. The nerve conduction chamber was built as an array of parallel metal pins, mounted at the side of an open coaxial cage (Fig. 2a). The proximal end of the isolated nerve was placed on the open central conductor of the coaxial line, and the length of the nerve was placed over the pins as shown in Fig. 2b. CAPs were elicited in the proximal site of the nerve, either by conventional electric stimuli (100–250 μs) from a Grass S88 Stimulator (Grass Instruments, Quincy, MA), or by 12-ns PEF from an FPG 20–1NM pulse generator (FID, Burbach, Germany). Both types of stimuli were applied through ground isolation units, a 4AD Stimulus Isolation Amplifier (Getting Instruments, San Diego, CA) and a PT-20k (Transient Plasma Systems, Torrance, CA), to eliminate ground loops and reduce recording artifacts. Conventional (long) stimuli were applied between pins 2 (anode) and 3 (cathode), Fig. 2b. For nsPEF stimulation, the leads for the conventional stimulation were physically disconnected, and pin 3 was connected to the shield (cathode) of the coaxial cage, while the central wire of the coaxial cable served as anode (Fig. 2b). This way, both stimulation modalities excited the nerve at the same location, at its contact with pin 3. CAPs traveled towards the distal end of the nerve and were recorded from the most remote pins, depending on the actual length of each nerve preparation; the other pins (between stimulation and recording sites) served for physical support and the amplifier ground connection. CAPs were recorded with a DAM50 differential amplifier (World Precision Instruments, Sarasota, FL) connected to a DSO5202P oscilloscope (Qingdao City, China) through a Humbug noise eliminator (Quest Scientific Instruments, North Vancouver, Canada). Monopolar CAP recording was accomplished by severing the nerve between two pins connected to the active leads of the amplifier. The entire length of the nerve was protected from drying with a Kwik-Cast Sealant (World Precision Instruments, Sarasota, FL).

An NSP50VI-6dB high voltage attenuator (Transient Plasma Systems) with a tap (1:100) for waveform monitoring was placed between the 12-ns PEF generator and the isolation unit. To minimize reflections, the 12-ns PEF line was closed on a NSP50VI-20dB high voltage attenuator (Transient Plasma Systems) and a 50 Ω load. The pulse amplitude and shape were continuously monitored with a 5-GHz TDS 3052 oscilloscope (Tektronix, Beaverton, OR). Nanosecond pulses (Fig. 2d) had a width of 12–14 ns at 50% height, 0.4–0.5 ns rise time (measured from 10% to 90% of the plateau amplitude), and an initial brief peak of 15–20% above the plateau.

### Electromagnetic characterization of the nerve conduction chamber

The quantification of the scattering parameters (S-parameters) of the conduction chamber and of the electric field distribution produced by 12-ns PEF was performed by numerical simulations with the commercial software HFSS ANSYS, Release 15.0 (ANSYS, Canonsburg, PA). Simulations were performed from 50 MHz to 1 GHz using an adaptive solution following the criterion of S-parameters optimization, which resulted in a mesh element minimum size of 0.006 mm, a maximum size of 26.9 mm, with 5,048,796 total number of elements in a volume of simulation of 3,840 cm^3^.

To quantify the electric field distribution produced by conventional pulses (steady-state conditions), we carried out 3D numerical simulations using a commercial finite element method solver COMSOL Multiphysics^®^, Release 5.0. The tetrahedral mesh chosen to discretize the domain of simulation resulted in a mesh element minimum size of 0.056 mm, a maximum size of 5.6 mm, and a total number of elements 3,303,214 in a volume of simulation of 11,494 cm^3^.

In both cases, the metal electrodes, the central wire, and the shield of the open coaxial cage were modeled as perfect electric conductors (PEC), while the plastic support as Plexiglas. The dimensions of the conduction chamber are shown in Fig. 2a.

Two port measurements of the S-parameters were carried out with an 8753E Vectron Network Analyzer, (VNA: Hewlett-Packard, Palo Alto, CA) in the frequency band from 30 kHz to 1 GHz.

In order for 12-ns PEF to travel undistorted from Port 1 to Port 2 of the conduction chamber, a good matching must be ensured up to ~100 MHz. Indeed, the spectrum of a trapezoidal 12-ns pulse (10 ns hold, 1 ns rise/fall time) has its first lobe at 92 MHz and the value at −3 dB at 40 MHz (Fig. 2d). Calculated modules of the reflection (|S_11_|) and transmission (|S_21_|) coefficients of the conduction chamber were below −10.3 dB and above −0.43 dB, respectively, up to 100 MHz (Fig. 2c, solid lines). Measurements of the S-parameters with the network analyzer confirmed modeling results (Fig. 2c, dashed lines).

**Figure 3:**
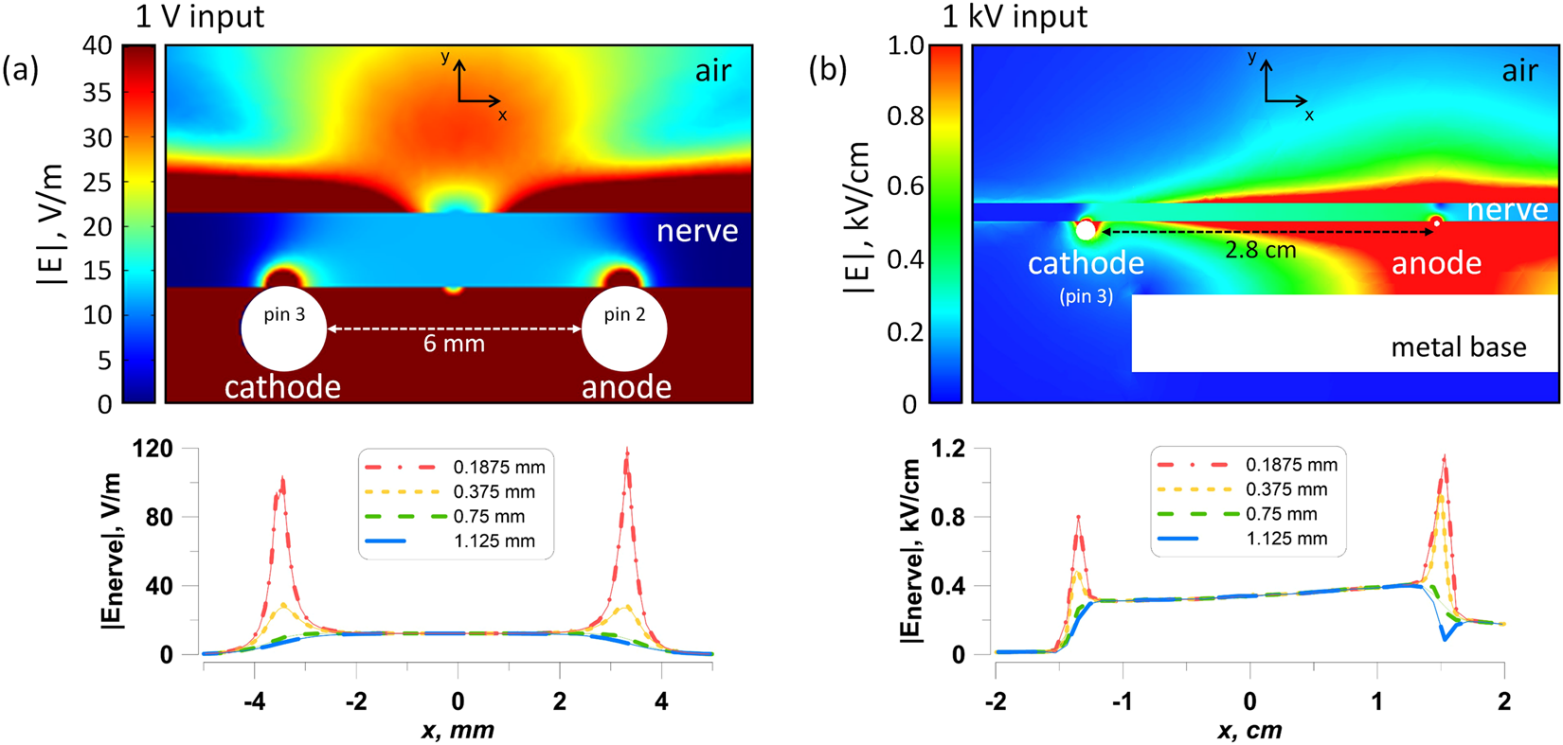
Electric field simulation results for conventional (**a**) and nsPEF (**b**) stimulation of the isolated nerve. Upper panels show the electric field distribution in a plane crossing the nerve perpendicular to stimulating electrodes. Note different values of the voltage input and different units for the electric field strength (1 V and V/m in **a**; 1 kV and kV/cm in **b**). Lower panels show the electric field values in the same plane, at four indicated depths into the nerve. For both conventional and nsPEF stimuli, action potentials in the nerve will be elicited at the cathode (pin 3). The electric field values reported elsewhere in this paper are the maximum values found over the cathode at the depth of 0.1875 mm into the nerve. See text for more details.

Simulations that account for the presence of the nerve (1.5 mm diameter) were carried out using isotropic dielectric properties^32^ (conductivity 0.6 S/m and relative permittivity 10^5^). The electric field distribution in the plane perpendicular to the nerve and stimulating electrodes is shown in Fig. 3a for conventional stimulation and Fig. 3b for 12-ns PEF when 1 V and 1 kV are applied between the electrodes, respectively. Two hot spots were present at the interface of the nerve with the anode and cathode. The lower panels show exact electric field values at 0.1875, 0.375, 0.75, and 1.125 mm from the electrodes.

For both conventional and nsPEF stimuli, action potentials in the nerve are elicited at the cathode (pin 3 in our setup). The depth of 0.1875 mm was considered sufficient to cross the nerve envelope and reach at least some of nerve fibers. Therefore, the electric field values reported elsewhere in this paper are the maximum values expected over the cathode at the depth of 0.1875 mm into the nerve (Fig. 3b, lower panels), namely 103 V/m per 1 V applied for conventional stimulation, and 0.8 kV/cm per 1 kV applied for nsPEF. The electric field intensity for nerve fibers located more remotely from the stimulating electrode (i.e., deeper into the nerve) could be times lower (Fig. 3).

Maximum heating (∆t, °C) of the nerve at the site of stimulation was estimated using an adiabatic heat equation^33^ as:

$${\rm{\Delta }}{\rm{t}}={1}{{0}}^{{3}}nt\sigma {E}^{2}/(\rho C),$$

where *σ* is the electrical conductivity (6.00 mS/cm), *ρ* is the density of the tissue (0.9 g/cm^3^), C is the heat capacity of nerve tissue (3.6 J/(g∙°C)), *E* is the local electric field in tissue (kV/cm), *t* is the pulse duration (s), and *n* is the number of pulses. For a typical tetanus protocol used (3,000 stimuli, 6.4 kV/cm), maximum heating without heat dissipation would be 1.8 °C. Even this maximum heating would not be damaging for a frog nerve, and the actual heating was smaller because of inevitable heat dissipation. However, transient thermal effects during the high-rate stimulation experiments cannot not be excluded.

### Data availability

The datasets generated during and/or analyzed during the current study are available from the corresponding author on reasonable request.

## Results

### Nerve response to a single 12-ns PEF

Each nerve preparation was first tested by conventional electrostimulation. Stimuli of 0.1–0.25 ms duration were applied at 3 Hz while tuning up the pulse amplitude. The threshold was found at 0.2–0.5 V, and the maximum CAP amplitude was reached at 1–3 V. For subsequent measurements, the pulse amplitude was set 20–50% higher, which ensured stable CAP recording for hours.

**Figure 4:**
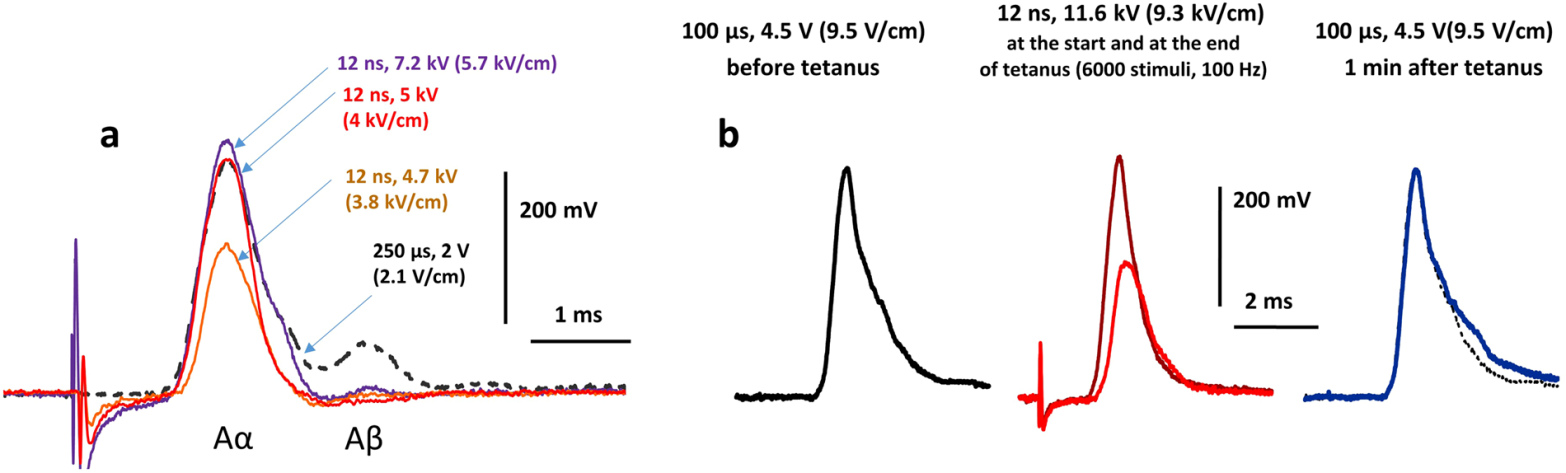
Compound action potentials (CAPs) evoked by conventional electrostimulation and by 12-ns PEF. (**a**) nsPEF elicit CAPs similar to conventional stimuli, but are selective for the excitation of fast fibers. Shown are CAP traces evoked by nsPEF at three different pulse voltages (solid lines) and by conventional stimulation (dashed line; stimulation parameters are given in the legends). Note that nsPEF at 7.2 kV elicits slightly larger response of the fastest fibers (Aα, first peak) than 250 µs, 2 V stimulation (330 and 300 µV, respectively) while the response of slower fibers (Aβ, the second peak) is 9-fold smaller (7 and 65 µV). (**b**) A 1-min, 100 stimuli/s tetanus using 12-ns, 11.6 kV pulses did not cause nerve damage. During the tetanus, CAP expectedly became smaller (central panel), but after a 1-min rest CAP evoked by conventional stimulation (right panel) was the same as before the stimulation (left panel; also shown by a dotted line in the right panel).

Next, the stimulator leads were disconnected and 12-ns pulses were applied, starting at 2.5 kV (the lowest output permitted by our setup). We found that the 12-ns stimuli can elicit CAP similarly to conventional stimuli, with the threshold of 4.1–6 kV (3.3–4.8 kV/cm). The CAP amplitude increased steeply and reached approximately the same maximum as with the conventional stimulation (Fig. 4a).

**Figure 5:**
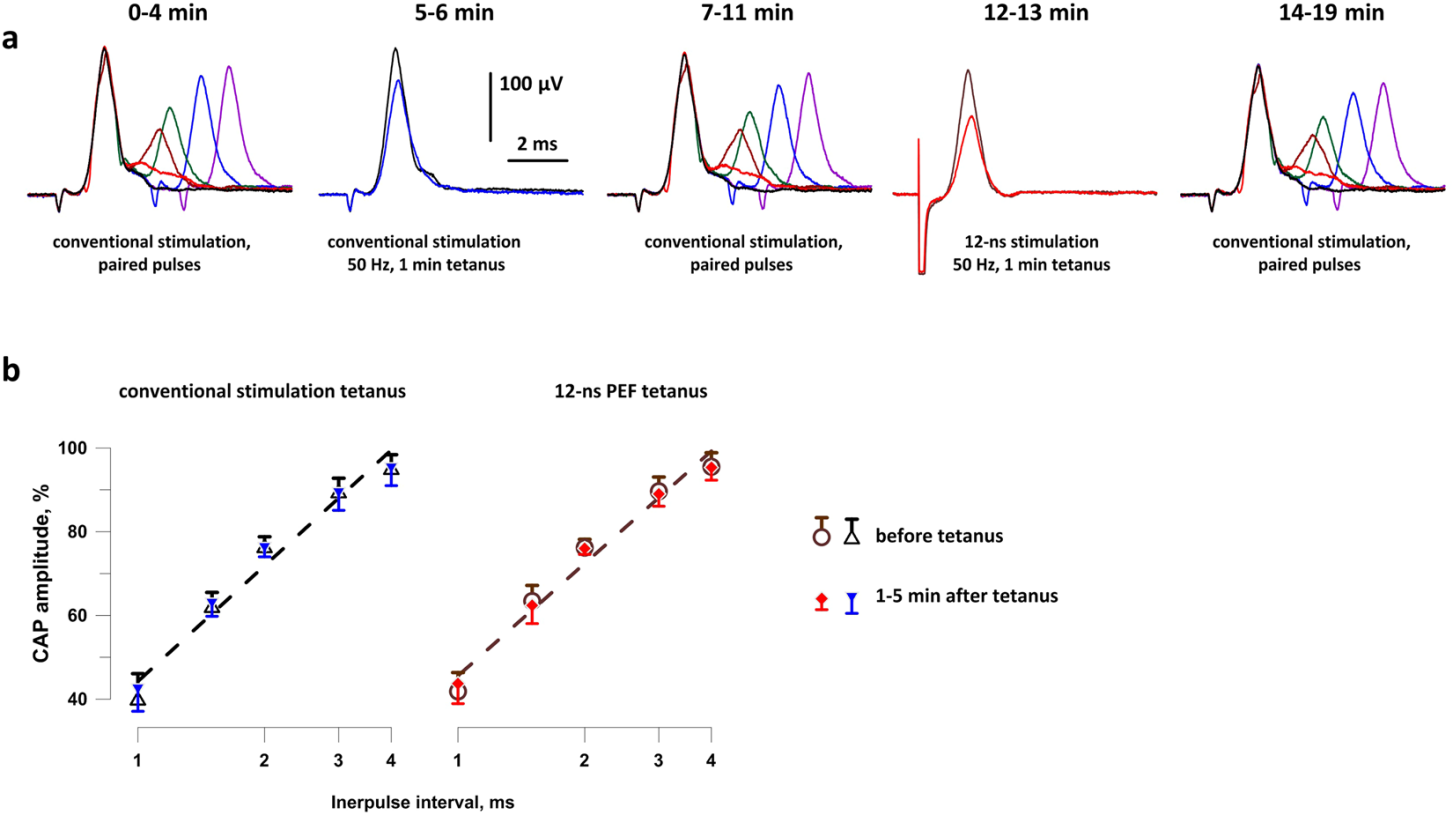
Tetanic stimulation (50 Hz, 1 min) by either conventional pulses or nsPEF does not change the nerve refractoriness. (**a**) The timeline and CAP traces from a representative experiment. Before and after each tetanus, CAPs were evoked by paired stimuli with interpulse intervals of 1, 1.5, 2, 3, and 4 ms. The tetani consisted of 3,000 single stimuli, either nsPEF (8 kV, 6.4 kV/cm) or conventional (2–4 V, 206–412 V/m), delivered with 50-ms intervals (1 min total). Shown in panels 2 and 4 are CAPs evoked by the first and the last stimuli in each tetanus (the latter CAP is smaller). See text for more details. (**b**) The inhibition of the test CAPs as a function of the interpulse interval, before and after tetani (open and filled symbols, respectively). The amplitude of the conditioning CAP is taken as 100%. Mean +/−  s.e., n = 8 for all groups. For clarity, error bars are shown in one direction only.

At the same time, nsPEF consistently showed lower stimulation efficiency for slower (thinner) nerve fibers. When the length of the nerve was sufficient to observe CAP dispersion, a separate excitation peak of slower fibers was present even before the stimulus amplitude became maximal for the CAP of the fastest fibers. In contrast, even supramaximal stimulation with nsPEF triggered little or no response of slower fibers (Fig. 4a,b; see also Fig. 5a). This phenomenon of selective excitation of faster fibers by nsPEF is intriguing from the mechanistic point of view and may be useful in many medical applications of electrostimulation; it will be addressed in detail in a separate paper.

### Excitation and electroporation damage to the nerve

As discussed in the Introduction, it is not clear how pulses as short as 12 ns (much shorter than VGSC activation time) can nonetheless trigger APs. One feasible mechanism would be the disruption of the nerve membrane by electroporation, leading to the loss of the membrane potential, eventual VGSC activation, and AP firing. The delay between the nsPEF and threshold depolarization of individual fibers would likely lead to somewhat less synchronized AP firing manifested by broadening of the CAP; however, no such broadening was observed (Fig. 4a).

Whenever electroporation occurs (including electroporation by nsPEF), its bioeffects can be enhanced by increasing either pulse voltage or pulse number^24, 33, 34^. Therefore, we tuned the voltage to the limit that could be sustained by our setup (8–12 kV) and applied large numbers of pulses as a more rigorous test for electroporative damage, as described below. In the past, applying multiple nsPEF stimuli helped to reveal electroporation effects which could not be observed with a single pulse^35, 36^ and was also reported to lower the threshold for detectable electroporation^33^.

Many conventional electroporation assays, such as propidium iodide uptake, are not suitable for the detection of membrane damage in the peripheral nerve: first, it does not contain enough nucleic acids for the detection of propidium entry and, second, it lacks transparency needed for live cell microscopy. Another hallmark of electroporation is a persistent loss of the resting membrane potential due to leak currents^24, 37^. In various types of cells, repair of the membrane and the restoration of the resting potential takes from tens of seconds to minutes^10, 17, 24, 37, 38^, consistent with data for other types of membrane injuries^39^. Then, repeated electroporating pulses which do not allow sufficient interval for the membrane repair would lead to cumulative damage and the complete loss of both the resting potential and fiber excitability. In other words, a high-rate tetanic stimulation can only be sustained if there is no electroporative injury incurred by each pulse.

Therefore, we subjected nerves to 1 min tetanus, either at 50 or 100 Hz (up to 11.6 kV, 9.3 kV/cm), and (a) monitored CAP during the tetanus and (b) compared the CAPs evoked by conventional stimulation before the tetanus and 1 min after it. It is well known that CAP amplitude gradually and reversibly decreases during a tetanus, which results from the reduction of the transmembrane ion gradient due to frequent AP conduction and is not a sign of damage. Figure 4b illustrates the gradual CAP diminution during the tetanus and its complete restoration just one minute after delivering as many as 6,000 nsPEF at the maximum voltage that could be achieved in our setup.

In many other experiments, there was a small difference in CAP amplitude measured before and after the tetanus, partly due to gradual deterioration of the nerve preparation *in vitro*. The peak amplitude and peak latency of CAP are reflective of both the number and the types of fibers that fired APs, and their changes reflect the engagement and rundown of the different fibers during tetanus. Thus, we compared the effects of 12-ns PEF tetani (50 Hz, 1 min, 8 kV, 6.4 kV/cm) with tetani of conventional stimuli (50 Hz, 1 min, 150–250 µs, 2–4 V, 206–412 V/m). The two treatments were applied repeatedly and alternated with each other. The peak amplitude and peak latency were measured in CAPs evoked by the conventional stimulation immediately before each tetanus (taken as 100%) and after a 1-min rest after the tetanus. We found that the different modalities of the tetanic stimulation decreased CAP amplitude to the same extent (to 90.0 ± 3.1% for nsPEF and to 94.5 ± 0.5% for conventional stimuli; *n* = 8, p > 0.1). However, the peak latency increased more after the nsPEF tetanus (by 4.0 ± 0.1% and by 0.6 ± 0.1%, respectively; p < 0.01), which could result from the different engagement of different types of fibers, as noted above.

Since nsPEF was applied locally to a small portion of the nerve at the stimulation electrode, it could not change the CAP conduction velocity between the stimulating and recording electrodes. The onset latencies of CAPs evoked by nsPEF and conventional stimuli were expectedly very similar and were not specifically compared in this study.

### No change in nerve refractoriness following nsPEF tetani

Refractory properties are arguably the most sensitive index of the physiological condition and any damages to the nerve. Therefore, we employed paired-pulse stimulation (with inter-pulse intervals of 1, 1.5, 2, 3, and 4 ms) to test if nsPEF and conventional pulse tetani affect the amplitude of CAPs elicited during the refractory period. This comparison was done in the same experiments as described above, but testing of the different interpulse intervals took 4–5 min extra time; such paired pulse stimulations preceded single pulse stimulation before each tetanus, and then were repeated at 1–5 min following the tetanus (Fig. 5a). The refractory period was always assessed by the conventional stimuli, while using either nsPEF or conventional stimuli for the tetanus.

These measurements established no effect of either conventional or nsPEF tetanic stimulation of the refractory properties of the nerve (Fig. 5), thus providing further proof that 12-ns PEF stimulation did not damage the nerve.

## Discussion

This is the first study which tested the ability of 12-ns electric pulses to stimulate a peripheral nerve. Stimulation was accomplished without damage to the nerve, despite applying pulse voltages as high as 8–11.6 kV (6.4–9.3 kV/cm). Each nerve preparation withstood tens of thousands of stimuli showing just slow rundown due to *in vitro* conditions and drying, but not associated with nsPEF impact. Our data prove that VGSC can be activated by voltage pulses as brief as 12 ns, although it remains to be elucidated if the activation is accomplished by the same steps as with the conventional electrostimulation. We did not observe the inhibition of VGSC that was earlier reported for longer 300-ns pulses when they were intense enough to cause membrane disruption^40^; the lack of VGSC inhibition confirms that nsPEF stimulation was accomplished without electroporative membrane damage.

The electric field thresholds for nerve excitation (>3.3 kV/cm at 12 ns pulse width) were in the same range as reported in earlier studies in the frog gastrocnemius muscle (24 kV/cm at 1.2 ns and 10 kV/cm at 100 ns) and in cardiomyocytes (>10 kV/cm at 4 ns^25^ and 36 kV/cm at 10 ns^11^). Some scattering of the data is expected and is likely a result of different pulse shapes and, in particular, of different methodologies employed for dosimetry. Importantly, these earlier studies tested only a few nsPEF in each sample and could not conclude with confidence whether nsPEF caused any damage. The very low thresholds reported for nociceptor neurons (0.13 kV/cm at 350 ns and 0.4 kV/cm at 12 ns^22, 23^) stand out and need independent confirmation.

In the next studies, we intend to build the strength-duration curves for different pulse durations and shapes in the nanosecond range. We will also focus on the selectivity of nsPEF for excitation of certain types of nerve fibers, which is particularly interesting from the mechanistic point of view and is promising for biomedical applications.
